# Relationship Between Inflammation and Metabolism in Patients With Newly Presenting Rheumatoid Arthritis

**DOI:** 10.3389/fimmu.2021.676105

**Published:** 2021-09-28

**Authors:** Gurpreet Singh Jutley, Kalvin Sahota, Ilfita Sahbudin, Andrew Filer, Thurayya Arayssi, Stephen P. Young, Karim Raza

**Affiliations:** ^1^ NIHR Birmingham Biomedical Research Centre, University Hospitals Birmingham NHS Foundation Trust and Institute for Inflammation and Ageing, University of Birmingham, Birmingham, United Kingdom; ^2^ Research Into Inflammatory Arthritis Centre, Versus Arthritis, University of Birmingham, Birmingham, United Kingdom; ^3^ Weill Cornell Medicine-Qatar, Education City, Doha, Qatar; ^4^ Department of Rheumatology, Sandwell and West Birmingham NHS Trust, Birmingham, United Kingdom

**Keywords:** inflammation, metabolism, rheumatoid arthritis, glycolysis, citrate cycle, urea cycle, oxidative stress, cachexia

## Abstract

**Background:**

Systemic inflammation in rheumatoid arthritis (RA) is associated with metabolic changes. We used nuclear magnetic resonance (NMR) spectroscopy–based metabolomics to assess the relationship between an objective measure of systemic inflammation [C-reactive protein (CRP)] and both the serum and urinary metabolome in patients with newly presenting RA.

**Methods:**

Serum (n=126) and urine (n=83) samples were collected at initial presentation from disease modifying anti-rheumatic drug naïve RA patients for metabolomic profile assessment using 1-dimensional ^1^H-NMR spectroscopy. Metabolomics data were analysed using partial least square regression (PLS-R) and orthogonal projections to latent structure discriminant analysis (OPLS-DA) with cross validation.

**Results:**

Using PLS-R analysis, a relationship between the level of inflammation, as assessed by CRP, and the serum (p=0.001) and urinary (p<0.001) metabolome was detectable. Likewise, following categorisation of CRP into tertiles, patients in the lowest CRP tertile and the highest CRP tertile were statistically discriminated using OPLS-DA analysis of both serum (p=0.033) and urinary (p<0.001) metabolome. The most highly weighted metabolites for these models included glucose, amino acids, lactate, and citrate. These findings suggest increased glycolysis, perturbation in the citrate cycle, oxidative stress, protein catabolism and increased urea cycle activity are key characteristics of newly presenting RA patients with elevated CRP.

**Conclusions:**

This study consolidates our understanding of a previously identified relationship between serum metabolite profile and inflammation and provides novel evidence that there is a relationship between urinary metabolite profile and inflammation as measured by CRP. Identification of these metabolic perturbations provides insights into the pathogenesis of RA and may help in the identification of therapeutic targets.

## Introduction

RA is a systemic inflammatory disease characterised by synovial inflammation and bone damage. Early RA appears to be a unique entity, with evidence that it is phenotypically distinct from established RA (1). Rapid initiation and escalation of treatment in early RA is associated with improved outcomes (2–4) and as such, the early stages of RA represent a unique “window of opportunity” (5). Systemic inflammation associated with early RA is responsible for significant extra-articular morbidity with an increased prevalence of stroke, heart failure (6), chronic obstructive pulmonary disease, asthma and interstitial lung disease (7) amongst early RA patients compared to matched controls. Furthermore, there is evidence of systemic changes in metabolism several years prior to onset of overt disease, which may be driven by early immune processes (8). Increased understanding of the relationship between inflammation and metabolism in early RA is thus important, in particular as therapies which target metabolic pathways emerge (9–11).

In RA, there is evidence of systemic immune activation and immune cell infiltration into synovium (12, 13). Synovial fibroblasts take on an aggressive inflammatory, matrix regulatory, and invasive phenotype. These fibroblasts, together with increased chondrocyte catabolism and synovial osteoclastogenesis, promote articular destruction (14, 15). In addition, inadequate lymphangiogenesis, which limits cell egress, together with local fibroblast activation, promotes the establishment of synovial inflammation. Immune cells involved in this inflammation are metabolically active (16). The resulting metabolic perturbations can lead to downstream effects (17).

Several metabolomics analyses of the serum and urine of patients with rheumatic diseases have been performed to date. A study of early inflammatory arthritis patients with a symptom duration of ≤3 months showed a relationship between CRP and the serum metabolome as assessed using NMR metabolomics with lactate and lipids as discriminators of inflammation (18). PLS-R models showed a relationship between the serum metabolome and CRP in two separate groups of early arthritis patients (r^2^ = 0.671, p<0.001 and r^2^ = 0.4157, p<0.001). Metabolomics has also been used to assess the relationship between low-grade inflammation and both the serum and the urinary metabolome in healthy individuals (19). Inflammation as measured by hsCRP was associated with multiple changes in metabolomes associated with oxidative stress and the urea cycle (19). Although urinary metabolomics has been shown to distinguish elevated disease activity in those with rheumatic diseases (20) and to predict responses to anti-TNF therapy in RA patients (21), urinary metabolomics has not been used to study the effect of inflammation as measured by CRP on metabolism in early RA before. However, findings from the serum metabolome are typically applicable to the urinary metabolome for instance the prediction of response to anti-TNF therapy in RA patients (21–23) and distinguishing healthy individuals with elevated inflammatory markers (19). Nevertheless, the relationship between the urinary metabolome and inflammation in patients with RA remains an understudied area.

We hypothesized that a metabolomics approach using serum, filtered to remove confounding high molecular weight species, and urine could identify a relationship between metabolic dysfunction and inflammation in patients with newly presenting RA.

## Material and Methods

### Patients

Patients were recruited from the Birmingham Early Arthritis Cohort (BEACON). BEACON includes patients presenting with DMARD naive inflammatory arthritis; details have been reported previously (16). This study focuses on patients with RA [classified using established criteria (24, 25)] recruited between January 2013 and September 2015. Patients with an unclassified arthritis (UA), recruited to BEACON during the same time period, were used as a non-RA inflammatory arthritis control group. All samples were defrosted and analysed by NMR spectroscopy at the same time, minimising magnetic field drift. Collectively, these considerations allow mitigation from some confounding factors while allowing assessment between systemic inflammation and metabolome in RA and a control inflammatory arthritis population.

The study was approved by the Black Country Research Ethics Committee and all patients gave written informed consent. The study was conducted over two sites: City Hospital, Sandwell and West Birmingham NHS Trust, Birmingham and Queen Elizabeth Hospital Birmingham, University Hospitals Birmingham NHS Foundation Trust. The following data were collected at baseline: age, gender, symptom duration, current medications, tender (26) and swollen (27) joint counts. Blood and urine were collected at presentation and processed as described below.

### Serum Samples

Samples of sera (RA; n=126 and UA; n=41) and urine (RA; n=83 and UA; n=25) were collected from patients at baseline, prior to initiating DMARD therapy. Blood was collected at presentation in vacutainer tubes containing clotting accelerator (Greiner Bio-one) and subsequently centrifuged at 600g for 10 minutes. Serum was removed and stored at minus 80°C until analysis. Serum samples were thawed at 4°C and centrifuged at 15,000g at 4°C for 5 minutes. To remove proteins, 200μl from the middle of the sample was placed into a Nanosep^®^ Omega 3000 Da (Pall Lifesciences, UK) molecular weight cut-off (MWCO) and centrifuged at 10,000g at 4°C for 15 minutes. Immediately prior to use, to remove the preservative glycerol, the filters were washed 6 times in distilled water at 37°C by centrifugation at 3000g for 15 minutes (28). The resulting filtrate was diluted in a 1 + 3 ratio with NMR buffer containing 1.6mM Difluorotrimethylsilylmethylphosphonic acid (DFTMP, Manchester Organics, Manchester, UK), 400mM phosphate, 40% D_2_O, 0.4% azide and 2mM 3-(Trimethylsilyl)-1-propanesulfonic acid-d6 sodium salt (DSS-d6, all from Merck, Southampton, UK). An aliquot (60ul) was removed to glass champagne vials (Cole-Parmer, Saint Neots, UK) and stored at -80°C until analysis.

### Urine Samples

Mid-stream urine samples were collected from patients at presentation to the clinic, centrifuged at 600g for 10 minutes and stored at minus 80°C. Samples were prepared using a standard protocol that has been used in other studies of urine (29). After thawing, urine samples (1ml) at 4°C were centrifuged at 15,000g for 5 minutes. A cleared sample (0.5ml) was mixed at 1:3 ratio with the 4x NMR buffer as for the serum above. The pH was adjusted (twice over a period of 30minutes) to pH 7.0. The samples were centrifuged at 15000g for 5 minutes and a sample (60ul) was removed to glass champagne vial and frozen at -80°C prior to NMR spectroscopy.

### NMR Spectroscopy

Samples were defrosted and transferred to 1.7mm NMR tubes (Bruker Biospin, Coventry, UK) using an Anachem Autosampler. After capping the tubes and wiping with dust-free paper, one-dimensional ^1^H spectra were acquired at 300K using a standard 1D-^1^H-Nuclear Overhauser Effect spectroscopy (NOESY) pulse sequence with water saturation using pre-sat in a Bruker AVANCE II 600 MHz NMR spectrometer (Bruker Corp., USA) equipped with a 1.7 mm cryoprobe. Spectral width was set to 12 ppm and the scans were repeated 128 times. Samples were loaded into racks and held at 6°C in the SampleJet sample handing device until processed. Two-dimensional 1H J-resolved (JRES) spectra were also acquired to aid metabolite identification (30).

Spectra were read and processed with Metabolab software (Version 2018.x; Birmingham, UK) (31). Each spectrum was phased according to the DSS-d6 peak, then aligned and corrected for baseline offset. The spectra were truncated to a range of 0.6 - 8.6 ppm (parts per million) and the water peak removed. Spectra were divided into chemical shift “bins” of 0.005 ppm and the spectral area of each bin integrated then scaled with probabilistic quotient normalization (PQN) to account for differences in sample dilutions (24) and normalised with a generalised log transform (λ = 1e^-08^) to equalize the weightings of smaller and larger peaks. Data were then compiled into a matrix where each row represented an individual sample before statistical analysis. Binning of spectra was performed as opposed to individual metabolite identification and quantification. This approach allows multivariate analysis on the entirety of the metabolomic data as opposed to on only the limited number of metabolites that can be definitively identified from the NMR spectra (25, 32).

### Statistical Analyses

#### Principal Components Analysis

The data bins from groups of spectra were mean centred and then assessed by PCA using Soft Independent Modeling of Class Analogy (SIMCA) version 14 (Umetrics) (33). PCA is an unsupervised multivariate mathematical analysis that extracts components in order of decreasing variance from multivariate datasets, enabling an understanding into the causes and effects behind these relationships.

#### Supervised Multivariate Analysis: Orthogonal Partial Least Square Discriminant Analysis (OPLS-DA) & Partial Least Square Regression (PLS-R) Analysis

Whilst PCA describes the relationship between possibly correlated variables in a single large multivariate matrix (matrix X) of data using PCs, partial least square is a multivariate analysis which attempts to describe the relationship between two different matrices of data using a latent variable (LV) approach to modelling the covariance in these two spaces. OPLS-DA was used to perform supervised clustering of samples using SIMCA version 14 (Umetrics) (33, 34). The OPLS-DA models were cross-validated using Venetian blinds (34), a method which reassigns randomly selected blocks of data to the OPLS-DA model to determine the accuracy of the model in correctly assigning class membership. The application of such methods to clinical studies is well established and guards against over fitting the model (35).

A PLS-R finds a linear regression model by projecting a predicted variable, which is created following application of an algorithm using latent variables to describe the covariance between the X and Y matrix, and the continuous variable in the Y matrix. Data bins were also subjected to PLS-R using the PLS Toolbox (version 5.8) (Eigenvector Research) in MatLab (release 2018b; MathWorks). This method identifies which metabolites can predict a continuous variable. This analysis yields an r^2^, a measure of the cross-validated goodness-of-fit of the linear regression, while permutation testing performed by multiple analyses using random data subsets, was used to assess the significance of this prediction. Models can be further optimised using a forward selection approach, which identifies a proportion of the metabolome that correlates with the continuous variable.

#### Identification of Metabolites & Pathway Analysis

Bins of interest, which may represent biomarkers, were identified for each statistically significant analysis. Weightings for each bin in PLS-R analysis models were assigned using regression coefficients Potential biomarkers were identified using +/- 2 standard deviations of the mean regression coefficient of the entire dataset (36). NMR spectra were annotated using Chenomx NMR suite (Chenomx, professional version 8.5) (37) programme. The Human Metabolome Database version 4.0 (38) and published lists of metabolites detectable by NMR spectroscopy of serum (25) and urine (32) were also used for labelling spectra.

Functional interpretation of the biomarkers implicated by the models was undertaken using MetaboAnalyst version 4 (39). A combination of both enrichment analysis and pathway analysis was used. Both analyses rely upon the identification of a metabolite as a biomarker, however they do not account for the direction of change of the metabolite. The enrichment analysis is an “over-representation” analysis. This tests whether a group of compounds involved in a pathway is enriched compared by random hits using a reference metabolome (40), thus are represented more than would be expected by chance. A hypergeometric test is used to generate a p value, which represents the probability of observing at least a specific number of metabolites from a certain metabolite set in a compound list. Pathway analysis incorporates both over representation analysis as discussed above and pathway topological analysis to determine which pathways are more likely to be involved by considering the pathway structure.

## Results

The baseline characteristics of patients included in the serum and urinary metabolomics analyses are shown in **Table 1**. PCA was used to generate an unbiased overview to investigate differences in metabolite profiles. OPLS-DA and PLS-R were used to perform supervised multivariate analyses. For both the PCA and OPLS-DA, a comparison was made between those individuals with low and high CRP values comparing patients in the lowest and highest CRP tertile groups. PLS-R analyses included all patients.

**Table 1:** Baseline characteristics of serum & urine metabolomics analysis of RA patients.

	RA patients included in sera metabolomics analysis (n = 126)	RA patients included in urinary metabolomics analysis (n = 83)	UA patients included in sera metabolomics analysis (n = 41)	UA patients included in urinary metabolomics analysis (n = 25)
Age, median (IQR) years	55 (47-62)	48 (55-60)	51 (42-60)	51 (38.5-60)
Missing (%)	0	0	0	0
Sex, no. (%) females	88 (69.8)	55 (66.3)	26 (63.4)	16 (64)
Missing (%)	0	0	0	0
Symptom duration, median (IQR) weeks	20.5 (11-47)	24 (12-45)	21 (12-42)	28 (14.5-50.5)
Missing (%)	0	0	0	0
CRP, median (IQR) mg/L	8 (3-16.3)	8 (3-16)	6 (3-21)	5 (3-11.5)
Missing (%)	0	0	0	0
DAS28CRP (IQR)	5 (4.3-5.8)	4.9 (4.2-5.7)	3.4 (2.7-4.5)	3.3 (2.7-4.4)
Missing (%)	2 (1.6)	2 (2.4)	0	0
RF positive, no. (%)	76 (60.3)	51 (61.4)	7 (17.1)	7 (28)
Missing (%)	0	0	0	0
ACPA positive, no. (%)	66 (52.4)	45 (54.2)	1 (2.4)	0 (0)
Missing (%)	0	0	0	0
NSAIDs, no. (%)	49 (38.9)	41 (49.4)	22 (53.7)	11 (44)
Missing (%)	0	0	0	0
Steroids, no. (%)	7 (5.6)	18 (21.7)	13 (31.7)	1 (4)
At baseline	3 (2.4)	3 (3.6)	2 (4.8)	0 (0)
Within last 3 months	4 (3.2)	15 (18.1)	11 (26.9)	1 (4)
Missing (%)	0	0	0	0

RA, Rheumatoid arthritis; IQR, Interquartile range; CRP, C reactive protein; DAS28CRP, Disease activity score 28 using C reactive protein; RF, rheumatoid factor; ACPA, anti-citrullinated protein antibody.

### Relationship Between Serum Metabolite Profile and CRP

**Figure 1:**
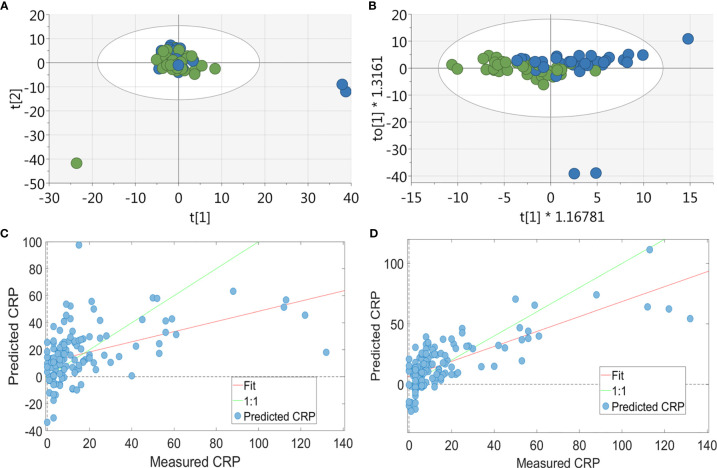
Multivariate analysis of RA patients’ serum metabolite profile. For the PCA & OPLSDA, patients were split into tertiles according to CRP values, with data shown for the highest and lowest tertile: **(A)** PCA plot of metabolic data derived from RA patients’ (n = 84) sera (green = CRP <5 and blue = CRP>13; 19 PC, r^2^ = 0.673) showing no separation between the two groups. **(B)** OPLS-DA plot of metabolic data derived from RA patients’ (n = 84) sera (green = CRP <5 and blue = CRP>13; 1 + 1+0 LV, p value= 0.033) showing a strong separation between the two groups. PLS-R analysis showed a relationship between serum metabolite profile and CRP. Using the full 590 serum metabolite binned data (n = 126) **(C)** there was a correlation between metabolite data and CRP on PLS-R analysis (r^2^ = 0.29, 7 LV, p < 0.001). Using forward selection, 36 bins were identified which correlated with inflammation and a subsequent PLS-R analysis using these bins **(D)** showed a stronger correlation between serum metabolite profile and CRP (r^2^ = 0.551, 6 LV, p = 0.001).

**Figure 2:**
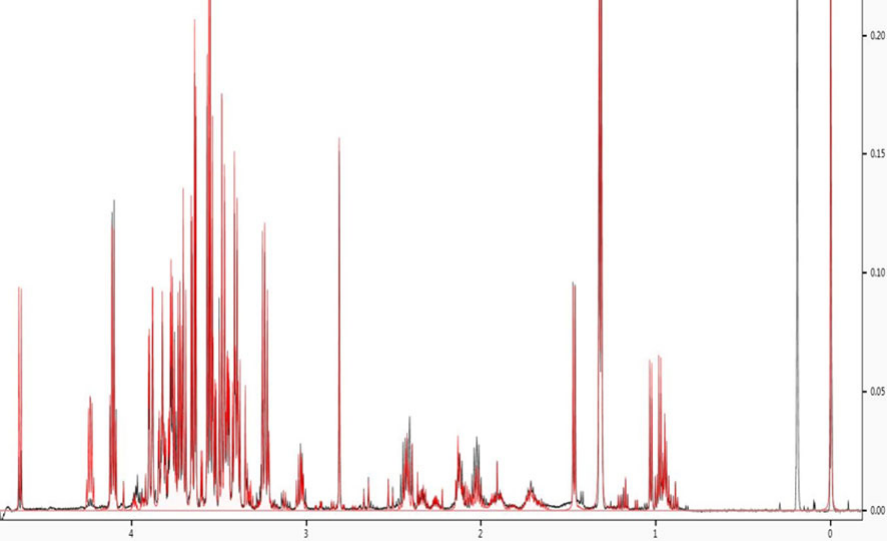
Spectral fitting to identify metabolites. NMR spectra were annotated using Chenomx NMR suite (Chenomx, professional version 8.5).

PCA showed no separation between patients in the lowest CRP tertile and the highest CRP tertile groups (**Figure 1A**). However, a supervised analysis using OPLS-DA showed a strong separation with 1 + 1+0 LV (**Figure 1B**; p=0.033). To investigate this further, the relationship between the serum metabolite profile and CRP was assessed using the regression analysis PLS-R. Using all 590 bins, a PLS-R analysis of metabolite data (**Figure 1C**) showed a statistically significant relationship between the serum metabolite profile and CRP (r^2^ = 0.29, 7 LV, p<0.001). Forward selection was carried out to produce a model containing the top 36 NMR bins (**Figure 1D**). This enhanced the relationship between metabolite profile and CRP (r^2^ = 0.551, 6 LV, p=0.001) compared to the original PLS-R. Spectral fitting to identify metabolites was performed using Chenomx (see **Figure 2**) and a published list of metabolites (25, 32). Potential metabolites identified by this model are shown in **Table 2**. Univariate analysis did not reveal a relationship between the concentrations of the metabolites identified in the bins with the three greatest regression coefficients (see **Table 2**) and CRP, except for citrate (R_s_=-0.302, p<0.001).

**Table 2:** Metabolites responsible for the relationship seen in PLS-R analysis between CRP and serum metabolite profile.

Order	Metabolite	Chemical shift of peak (ppm)	Regression coefficient
1	Citrate	2.534, 2.5048, 2.6511, 2.5106, 2.6745, 2.5282, 2.6394, 2.6453, 2.6886, 2.6687	↓↓↓↓↓↓↓↓↓↓
1	Glutamine	2.534, 2.5048, 2.5106, 2.5282, 2.3643, 2.3701, 2.0846, 3.6813	↓↓↓↓↑↑↑↓
2	Lactate	1.2585, 1.2349, 1.1116, 1.3633, 1.4921	↑↓↑↑↓
2	Threonine	1.2585, 1.2349, 1.3633, 3.6111	↑↓↑↓
2	Isoleucine	1.2585, 1.2349, 1.4921, 0.89505, 3.6813	↑↓↓↑↓
3	Glucose	5.1799, 2.7916, 3.1948, 5.174, 2.8911, 2.7858, 3.6111, 3.336, 3.3418, 3.6813, 2.8741, 3.1253	↑↑↓↑↑↑↓↑↑↓↑↓
4	Pyruvate	2.5048, 2.5106, 2.6687	↓↓↓
5	Aspartate	2.7916, 2.6511, 2.6745, 2.6394, 2.6453, 2.7858, 2.6886	↑↓↓↓↓↑↓
5	Methylguanidine	2.7916, 2.8911, 2.7858, 2.8741	↑↑↑↑
6	Formate	8.4462	↑
7	Carnitine	3.1948, 3.336, 3.3418, 3.1253	↓↑↑↓
7	Glycerol	3.1948, 3.6111, 3.336, 3.3418, 3.6813, 3.1253	↓↓↑↑↓↓
7	Betaine	3.1948, 3.336, 3.3418	↓↑↑
7	3-methylhistidine	3.1948, 7.0472, 3.336, 3.6813	↓↓↑↓
7	Arginine	3.1948, 3.336, 1.7482	↓↑↓
7	Tyrosine	3.1948, 7.1291, 7.1463, 3.1253	↓↑↑↓
7	Cystine	3.1948, 3.336, 3.3418	↓↑↑
7	Choline	3.1948	↓
8	Methionine	2.6511, 2.6394, 2.6453, 2.0846, 2.6687	↓↓↓↑↓
9	3-hydroxybutyrate	1.2349	↓
9	Isopropanol	1.2349	↓
10	Asparagine	2.8911, 2.8741	↑↑
11	Phenylalanine	7.3164, 7.3223, 3.1253, 7.4042, 7.4159, 7.3106	↑↑↓↑↑↑
12	Histidine	7.0472, 3.1253	↓↓
13	Proline	2.3643, 2.3701, 3.336, 3.3418, 2.0846	↑↑↑↑↑
13	Succinate	2.3643, 2.3701	↑↑
13	Glutamate	2.3643, 2.3701, 2.0846	↑↑↑
14	Valine	1.1116, 3.6111, 0.89505	↑↓↑
14	Propylene glycol	1.1116	↑
15	Alanine	1.3633, 1.4921	↑↓
15	Lysine	1.3633, 1.4921, 3.6813, 1.7482, 3.1253	↑↓↓↓↓
16	Glycine	3.6111	↓
17	Methanol	3.336, 3.3418	↑↑
18	2-hydroxybutyrate	0.89505, 1.7482	↑↓
18	Leucine	0.89505, 3.6813, 1.7482	↑↓↓
19	Ornithine	1.7482	↓
20	Malonate	3.1253	↓
20	Cysteine	3.1253	↓
21	Tryptophan	7.3106	↑

The following metabolites have been ranked by the magnitude of the regression coefficient. The bins that each metabolite was implicated as a biomarker were also listed by descending order of magnitude of regression coefficient. The regression coefficient field indicates the nature of correlation (↑ indicating a positive relationship with CRP and ↓ indicating a negative relationship with CRP).

**Figure 3:**
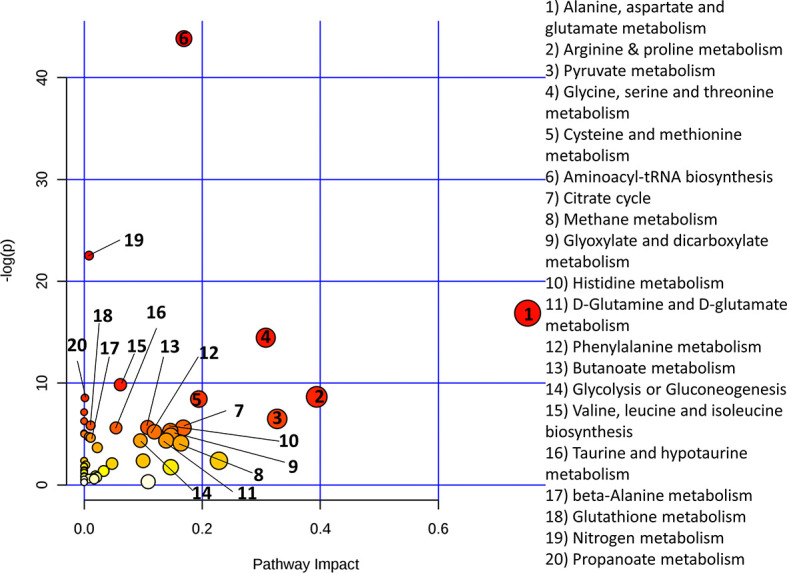
Metaboanalyst pathway analysis of potential biomarkers implicated by PLS-R analysis of CRP and patients’ serum metabolites.

**Figure 4:**
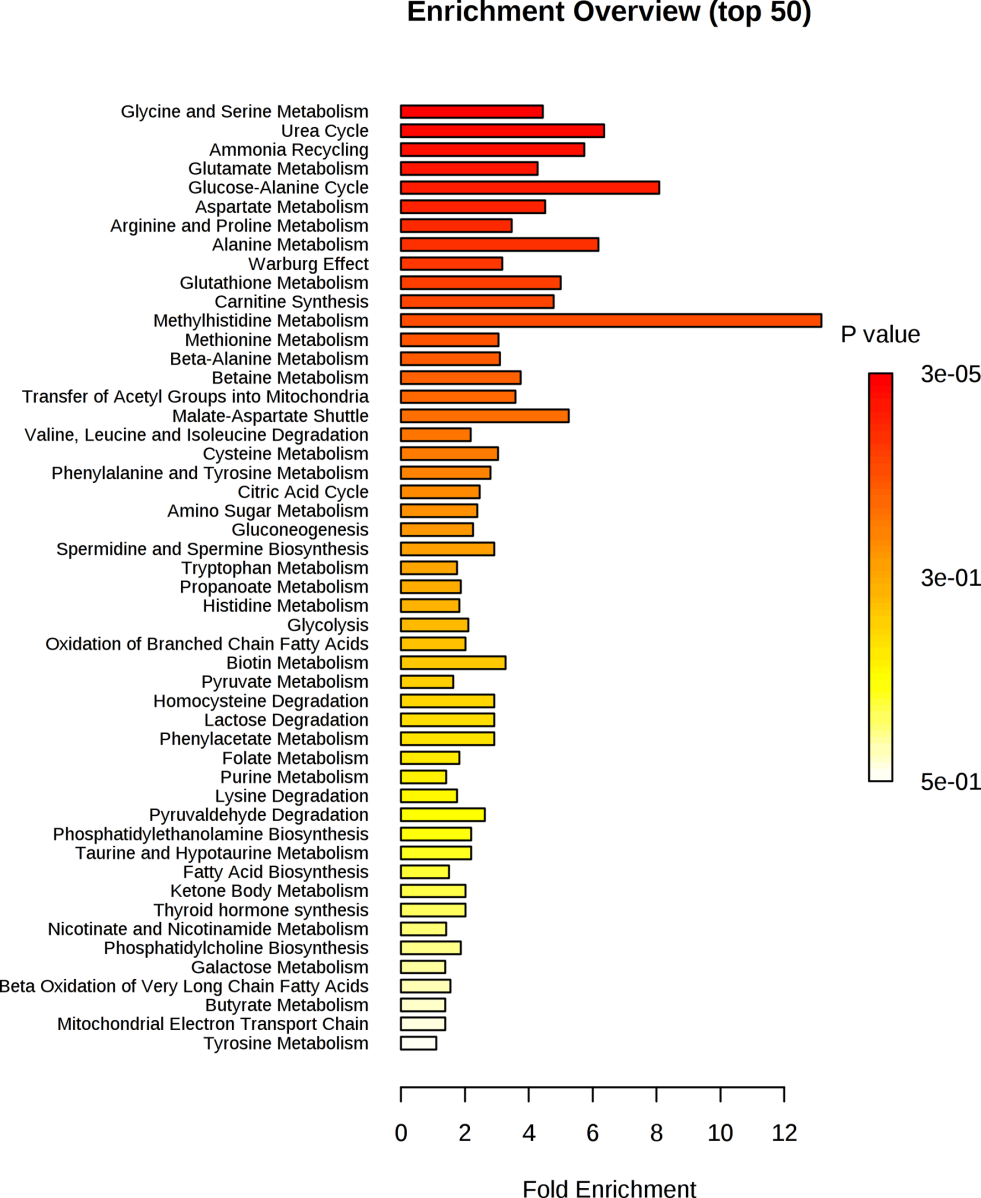
Enrichment analysis of key metabolites in serum implicated as potential biomarkers by the PLS-R analysis of CRP and patients’ serum metabolites.

Functional metabolomics analysis based on the biomarkers identified by PLSR analysis showed alanine, aspartate and glutamate metabolism, arginine and proline metabolism, pyruvate metabolism and glycine, serine and threonine metabolism are altered in the serum of RA patients with elevated CRP (**Figure 3**). Over-representation analysis (**Figure 4**) in pathway-associated metabolite sets indicated that amongst the multiple pathways which were implicated, methylhistidine metabolism, the urea cycle and the glucose alanine cycle were the most overrepresented in the serum of patients with elevated CRP. These results suggested that perturbed energy and amino acid metabolism in the serum are key characteristics of RA patients with elevated CRP.

### Relationship Between Urinary Metabolite Profile and CRP

**Figure 5:**
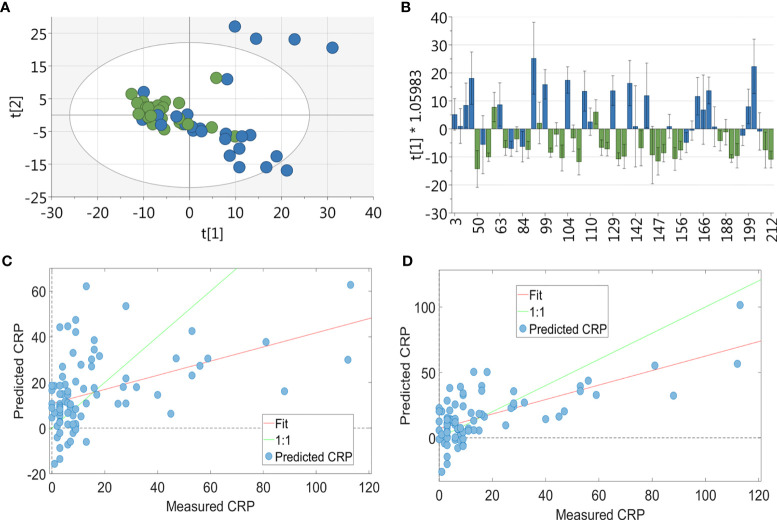
Multivariate analysis of RA patients’ urinary metabolite profile. For the PCA & OPLSDA, patients were split into tertiles according to CRP values, with data shown for the highest and lowest tertile (n = 54): **(A)** PCA plot of metabolic data derived from RA patients’ urine (green = CRP <5 and blue = CRP>11; 19 PC, r^2^ = 0.673) showing no separation between the two groups. **(B)** OPLS-DA plot of urinary metabolic data (n = 83, green = CRP <5 and blue = CRP>11; 1 + 0+0 LV, p value < 0.001) showing a strong separation between the two groups. PLS-R analysis showing the relationship between urinary metabolites and CRP. Using the full 900 NMR urinary metabolite bins for RA patients (n = 83) **(C)** there was a correlation between metabolite profile and CRP (r^2^ = 0.095, 1 LV, p = 0.008). Using forward selection, 144 bins were identified which most strongly correlated with CRP and a subsequent PLS-R using these bins **(D)** showed a correlation between urinary metabolite profile and CRP (r^2^ = 0.429, 3 LV, p < 0.001).

PCA was used to generate an unbiased overview to identify differences between patients in the lowest CRP tertile and the highest CRP tertile (**Figure 5A**). There was no discernible separation between these groups. However, a supervised analysis using OPLS-DA (**Figure 5B**) showed a strong separation with 1 + 0+0 LV (p value<0.001). Using all 900 bins, PLS-R analysis (**Figure 5C**) showed a correlation between urinary metabolite profile and serum CRP (r^2^ = 0.095, 1 LV, p=0.008). Using a forward selection approach, a PLS-R using 144 urinary NMR bins (**Figure 5D**) produced the most optimal correlation with CRP (r^2^ = 0.429, 3 LV, p<0.001). Metabolites identified by this model are shown in **Table 3**. Univariate analysis assessing the relationships between CRP and the concentrations of the metabolites identified in the bins with the three greatest regression coefficients (see **Table 3**) showed a relationship between CRP and 3-aminoisobutyrate (R_s_=0.504, p=0.001), alanine (R_s_=0.302, p=0.004), cystathionine (R_s_=0.579, p<0.001), phenylalanine (R_s_=0.593, p<0.001), cysteine (R_s_=0.442, p=0.003), and 3-methylhistidine (R_s_=0.383, p<0.001) respectively.

**Table 3:** Metabolites responsible for the relationship seen in PLS-R analysis between CRP and urinary metabolite profile.

Order	Metabolite	Chemical shift of peak (ppm)	Regression coefficient
1	3-Aminoisobutanoic acid	1.1431, 1.1376, 1.1303, 1.127, 3.0865, 1.0904, 1.1019, 1.1607, 3.0923, 1.1468, 1.1665, 3.0982, 1.155, 3.104	↓↓↓↓↓↑↑↓↓↓↓↓↓↓
1	Propylene glycol	1.1431, 1.1376, 1.1303, 1.127, 1.1468	↓↓↓↓↓
2	Lysine	1.4943, 1.4885, 1.506, 1.5002, 1.4416	↑↑↑↑↑
2	Azelaic acid	1.4943, 1.4885, 1.506, 1.5002, 2.1733	↑↑↑↑↓
2	Sebacic acid	1.4943, 1.4885, 1.506, 1.5002, 2.1733	↑↑↑↑↓
2	3-Methyladipic acid	1.4943, 1.4885, 1.506, 1.5002, 2.1733	↑↑↑↑↓
2	Suberic acid	1.4943, 1.4885, 1.506, 1.5002, 2.1733	↑↑↑↑↓
2	Alanine	1.4943, 1.4885, 1.506, 1.5002, 1.4416	↑↑↑↑↑
2	3-Methyl-2-oxovaleric acid	1.4943, 1.1303, 1.127, 1.4885, 1.0904, 1.1019, 1.5002, 1.4416, 1.0558	↑↓↓↑↑↑↑↑↓
3	L-Cystathionine	3.0865, 3.0923, 3.0982, 2.7353, 2.7294, 2.1733, 2.706, 2.7587, 3.104, 3.8474, 3.8416	↓↓↓↑↑↓↑↑↓↓↓
3	Creatinine	3.0865, 2.7879, 3.0923, 2.8172, 3.0982, 2.7353, 2.7294, 3.1567, 2.8406, 2.706, 2.7587, 2.7938, 3.104	↓↑↓↑↓↑↑↓↑↑↑↑↓
3	Phenylalanine	3.0865, 3.0923, 7.2191, 3.0982, 7.3819, 7.2484, 7.5001, 7.2425, 7.225, 7.3771, 3.104, 7.4123	↓↓↑↓↑↑↑↑↑↑↓↓
3	Cysteine	3.0865, 3.0923, 3.0982, 3.104	↓↓↓↓
3	3-methylhistidine	3.0865, 7.7518, 3.0923, 3.1567	↓↑↓↓
4	2-Ketobutyric acid	1.0904, 1.1019, 2.7879, 2.7587, 1.0558	↑↑↑↑↓
4	Methylsuccinate	1.0904, 1.1019, 1.0558	↑↑↓
5	Hippuric acid	7.7518, 7.9918, 7.7768, 7.9332, 7.8513, 7.5001, 7.9976, 7.781	↑↓↑↑↑↑↓↑
5	Tryptophan	7.7518, 7.2484, 7.5001, 7.225	↑↑↑↑
5	Phenylglyoxylic acid	7.7518, 7.9918, 7.7768, 7.9497, 7.9332, 7.9976, 7.781, 7.947	↑↓↑↑↑↓↑↑
5	Urea	7.7518, 7.9918, 7.7768, 7.2191, 7.3819, 7.9497, 7.9332, 7.2484, 7.5001, 7.9976, 7.781, 7.2425, 7.225, 7.3771, 7.947	↑↓↑↑↑↑↑↑↑↓↑↑↑↑↑
5	7-Methylxanthine	7.7518, 7.9918, 7.7768, 7.9332, 7.8513, 7.9976, 7.781, 3.8474, 3.8416	↑↓↑↑↑↓↑↓↓
6	Dihydrothymine	1.1607, 2.7879, 1.1665, 3.1567, 1.155, 2.7587, 2.7938	↓↑↓↓↓↑↑
7	Quinolinic acid	7.9918, 7.9976	↓↓
7	Carnosine	7.9918, 2.7294, 3.1567, 7.9976, 2.706	↓↑↓↓↑
7	Picolinic acid	7.9918, 7.8981, 7.9497, 7.9332, 7.9976, 7.947	↓↑↑↑↓↑
7	Histidine	7.9918, 7.8981, 7.9332, 3.1567, 7.9976	↓↑↑↓↓
8	Succinylacetone	2.7879, 2.8172, 2.8406, 2.7938, 3.8474, 3.8416	↑↑↑↑↓↓
8	Aspartate	2.7879, 2.8172, 2.8406, 2.7938	↑↑↑↑
8	Methylguanidine	2.7879, 2.8172, 2.7938	↑↑↑
8	Citrate	2.7879, 2.8172, 2.7353, 2.7294, 2.8406, 2.706, 2.7587, 2.7938	↑↑↑↑↑↑↑
8	5-Aminolevulinic acid	2.7879, 2.7587, 2.7938	↑↑↑
8	Levulinic acid	2.7879, 2.7587	↑↑
9	Malonate	3.0923, 3.0982, 3.104	↓↓↓
10	Symmetric dimethylarginine	2.8172	↑
11	4-Hydroxybenzoic acid	7.7768, 7.781	↑↑
12	Indoleacetate	7.2191, 7.2484, 7.5001, 7.2425, 7.225	↑↑↑↑↑
13	trans-Ferulic acid	7.2191, 7.225	↑↑
13	Tyrosine	7.2191, 7.225	↑↑
13	Ortho-Hydroxyphenylacetate	7.2191, 7.225	↑↑
13	Indoxyl sulfate	7.2191, 7.3819, 7.5001, 7.225, 7.3771	↑↑↑↑↑
13	Tryptophan	7.2191	↑
13	Phenylacetate	7.2191, 7.3819, 7.2484, 7.2425, 7.225, 7.3771	↑↑↑↑↑↑
14	Mandelic acid	7.3819, 7.3771, 7.4123	↑↑↓
15	Cinnamic acid	7.3819, 7.5001, 7.3771, 7.4123	↑↑↑↓
16	Cystine	3.3792, 3.1567, 3.385	↓↓↓
16	4-Hydroxyproline	3.3792, 2.1733, 3.385	↓↓↓
16	Pantothenic acid	3.3792	↓
17	Anserine	2.7353, 2.7294, 2.706	↑↑↑
17	Sarcosine	2.7353, 2.7294, 2.7587	↑↑↑
17	Citramalic acid	2.7353, 2.7587	↑↑
18	Kynurenic acid	7.8981, 7.9332, 7.5001	↑↑↑
18	3-Methylhistidine	7.8981, 7.9332	↑↑
19	Benzoic acid	7.9332, 7.8513, 7.5001	↑↑↑
20	3-Hydroxyphenylacetate	7.2484, 7.2425, 7.225	↑↑↑
21	L-Kynurenine	7.8513, 7.4123	↑↓
22	Ethanolamine	3.1567, 3.8474, 3.8416	↓↓↓
22	Beta-Alanine	3.1567	↓
23	Asparagine	2.8406	↑
24	Vanillic acid	7.5001	↑
24	Uracil	7.5001	↑
24	4-Pyridoxic acid	7.5001	↑
24	Cytosine	7.5001	↑
25	Monomethyl glutaric acid	2.1733	↓
25	Pimelic acid	2.1733	↓
25	Methionine	2.1733, 3.8474, 3.8416	↓↓↓
25	Isovalerylglycine	2.1733	↓
25	Glutamate	2.1733	↓
25	Methylglutaric acid	2.1733	↓
25	L-2-Hydroxyglutaric acid	2.1733	↓
25	Glutamine	2.1733	↓
26	Isoleucine	1.4416	↑
27	Dihydrouracil	2.706	↑
28	Valine	1.0558	↓
29	L-Arabitol	3.8474, 3.8416	↓↓
29	Serine	3.8474, 3.8416	↓↓
29	N-Acetylneuraminic acid	3.8474, 3.8416	↓↓
29	D-Maltose	3.8474, 3.8416	↓↓
29	Pseudouridine	3.8474, 3.8416	↓↓
29	Thymidine	3.8474, 3.8416	↓↓
29	Hydroxypropionic acid	3.8474, 3.8416	↓↓
29	Alpha-Lactose	3.8474, 3.8416	↓↓
29	Adenosine	3.8474, 3.8416	↓↓
29	Sorbitol	3.8474, 3.8416	↓↓
29	D-Galactose	3.8474, 3.8416	↓↓
29	Homovanillic acid	3.8474, 3.8416	↓↓
29	D-Xylitol	3.8474, 3.8416	↓↓
29	Gluconic acid	3.8474, 3.8416	↓↓
29	L-Arabinose	3.8474, 3.8416	↓↓
29	Sucrose	3.8474, 3.8416	↓↓
29	Dehydroascorbic acid	3.8474, 3.8416	↓↓
29	1-Methyladenosine	3.8474, 3.8416	↓↓
29	Glyceric acid	3.8474, 3.8416	↓↓

The following metabolites have been ranked by the magnitude of the regression coefficient. The bins that each metabolite was implicated as a biomarker were also listed by descending order of magnitude of regression coefficient. The regression coefficient field indicates the nature of correlation (↑ indicating a positive relationship with CRP and ↓ indicating a negative relationship with CRP).

**Figure 6:**
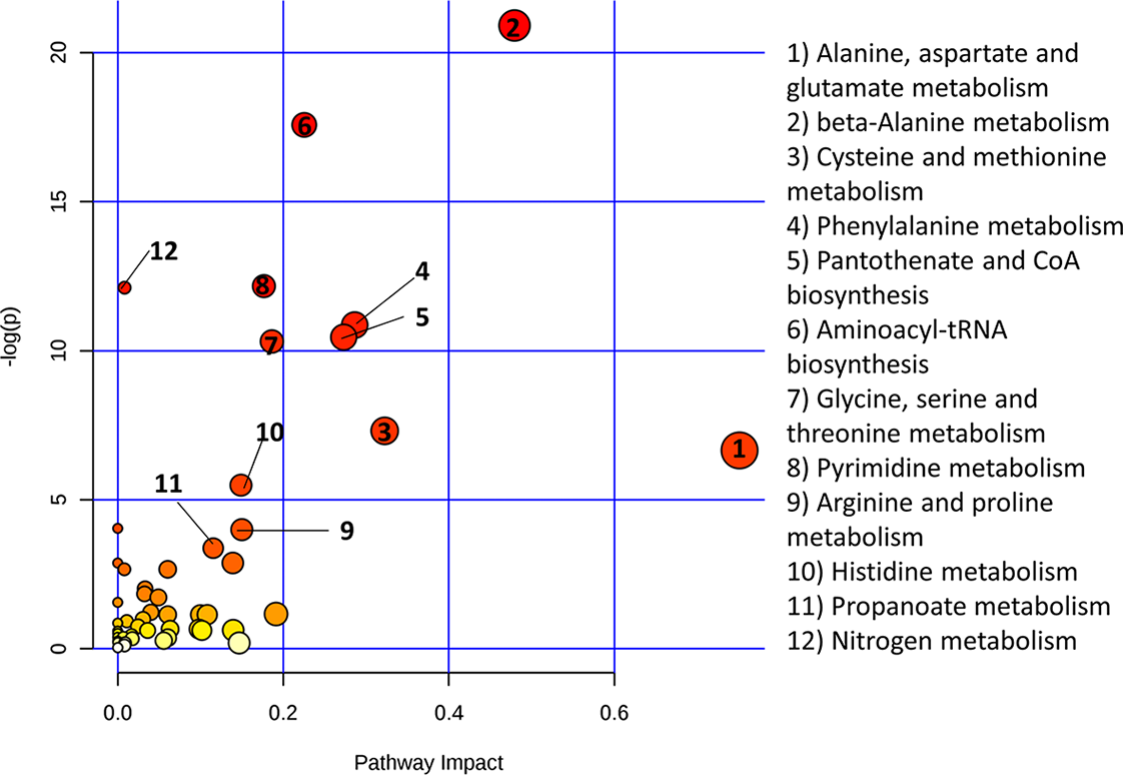
Metaboanalyst pathway analysis of potential biomarkers implicated by PLS-R analysis of CRP and patients’ urinary metabolites.

**Figure 7:**
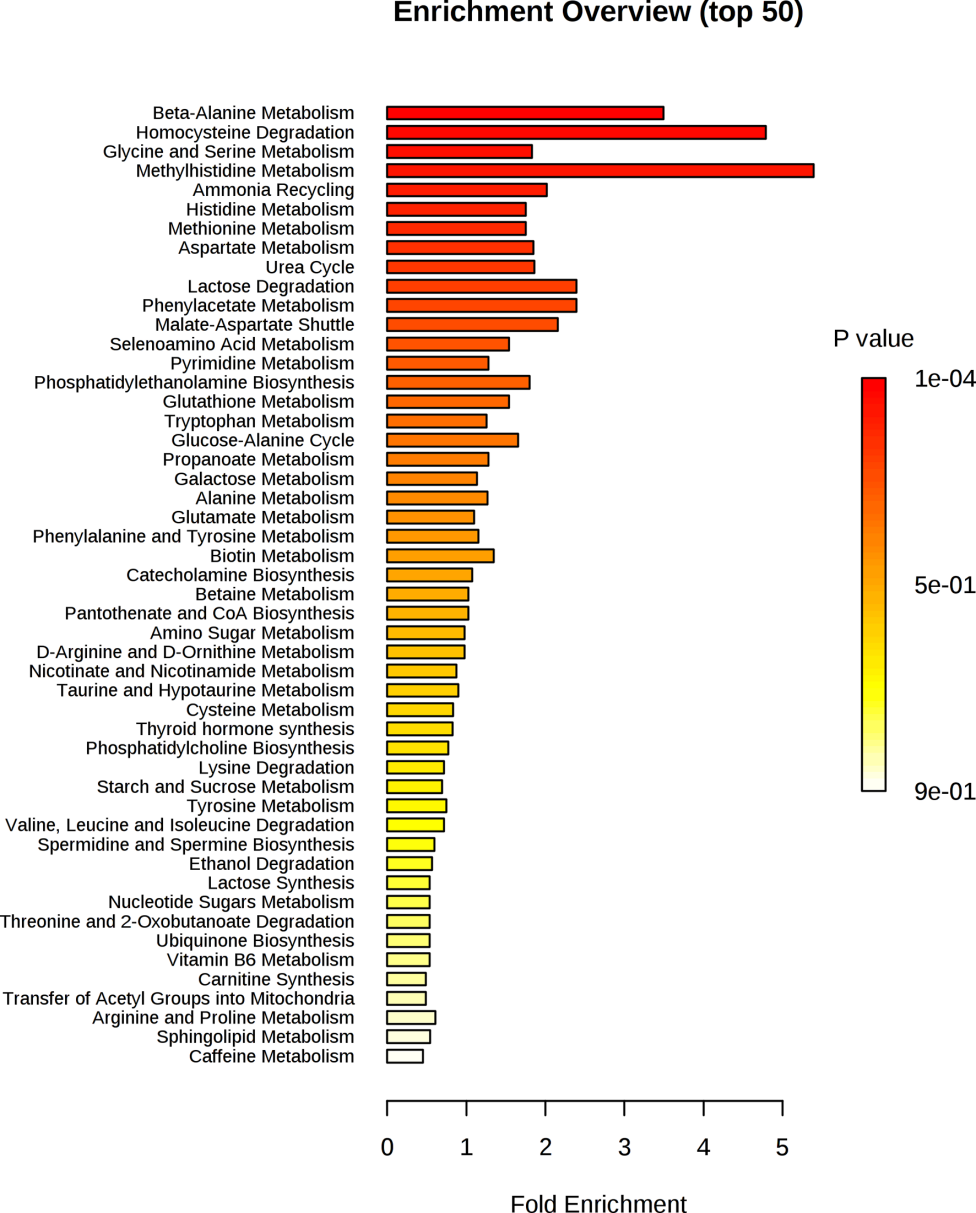
Enrichment analysis of key metabolites in urine implicated as potential biomarkers by the PLS-R analysis of CRP and RA patients’ urinary metabolites.

**Figure 6** shows that alanine, aspartate and glutamate metabolism and beta-alanine metabolism were the most impacted metabolic pathways. **Figure 7** shows the enrichment analysis using metabolites identified by PLS-R analysis of RA patients’ urinary metabolite data and CRP. Beta-alanine metabolism, glycine and serine metabolism, homocysteine degradation and methylhistidine metabolism were the only overrepresented metabolic pathways that reached statistical significance.

Further analyses assessed the correlations between metabolic data derived from RA serum/urine and ESR (**Supplementary Figure 1**), autoantibody status (**Supplementary Figures 2A, B**) and symptom duration (**Supplementary Figures 2C, D**). PLS-R analysis showed a correlation between serum metabolite data and ESR (n=120, r^2^ = 0.15, 5 LV, p=0.013). Likewise, a PLS-R analysis showed a correlation between urinary metabolite data and ESR (n=79, r^2^ = 0.19, 5 LV, p=0.014). OPLS-DA showed no separation between seropositive (for either ACPA or RF or both) and seronegative RA patients based on either serum (p=1) or urinary (p=1) metabolic data. Additionally, OPLS-DA showed no separation between early (symptom duration of ≤12 weeks) and established (symptom duration of >12 weeks) RA patients based on either serum (p=0.556) or urinary (p=1) metabolic data.

In order to assess whether the relationship between the metabolome and CRP was specific to RA or was seen in non-RA inflammatory arthritis, serum and urine were analysed from patients with UA. Similar to the correlations between CRP and metabolic data derived from RA patients’ serum and urine samples, a relationship was also seen between CRP and metabolic data derived from the sera (n=41, r^2^ = 0.7209, 9 LV, p<0.001) and urine (n=25, r^2^ = 0.6117, 8 LV, p=0.025) of UA patients (**Supplementary Figure 3**).

## Discussion

In this study, we applied ^1^H-NMR metabolomics to assess the relationship between systemic inflammation, as assessed by the serum CRP, and the serum and urinary metabolome in a group of DMARD naïve newly presenting RA patients. Young et al. (18) have previously shown a significant relationship between metabolites identified in unfiltered serum and CRP in two groups of early inflammatory arthritis patients. The metabolites which contributed to that relationship included low-density lipoprotein lipids, lactate, glucose, methylguanidine and amino acids and their derivatives (taurine, acetylglycine, choline, threonine and methylhistidine) (18). Furthermore, a relationship between CRP, measured using a high sensitivity assay, and metabolites in plasma and urine of healthy individuals has been previously seen, with permutations of metabolites related to oxidative stress and the urea cycle observed (19).

In the present study, filtered serum was used which is devoid of large proteins and lipoproteins. This was done to avoid the significant overlap of the broad NMR signals of proteins and lipoproteins with the metabolites in the spectra (41), which can lead to difficulty in identifying individual metabolites. Despite losing information provided by proteins and lipoproteins, filtration of serum results in spectra with fewer overlapping metabolites which can make metabolite identification less problematic. Loss of lipoproteins notwithstanding, PLS-R analysis of filtered serum identified a significant relationship between serum metabolites and CRP (r^2^ = 0.551, 6 LV, p=0.001). The most highly weighted metabolites in the model included glucose, amino acids, lactate, and citrate. This validates the previously identified relationship between metabolites and CRP (18). Furthermore, it shows a definitive relationship between CRP and metabolites which persist in filtered serum.

Our study also showed a relationship between urinary metabolites and CRP. Blood concentrations of metabolites are strictly regulated, while urine metabolite concentrations can vary widely and can provide complementary information about systemic metabolism. In addition to filtration, the kidney has important role in the generation, breakdown, and active reabsorption and secretion of metabolites, which together determine urinary metabolite concentrations (42, 43). Urinary metabolomics has previously been used to predict responses to anti-TNF treatment in patients with RA (21) and to facilitate diagnosis (20, 44) in patients with inflammatory rheumatic conditions. Pietzner et al. demonstrated a serum and urinary metabolic signature of chronic low grade inflammation in apparently healthy individuals (19). Our findings extend this observation showing a relationship between clinically apparent inflammatory states and the urinary metabolome. The functional interpretation of biomarkers generated by PLS-R analysis largely confirmed the findings seen in the serum analysis, namely increased urea cycle activity, oxidative stress and protein catabolism.

In addition to showing a relationship between CRP and metabolic data derived from RA patients’ serum and urine samples, our study showed a relationship between ESR and metabolic data derived from RA patients’ serum and urine samples. Furthermore, we were able to show the relationship between CRP and metabolome is not specific to RA but is also present in non-RA inflammatory arthritis. This suggests the relationship between inflammation and the metabolome is present independent of the underlying of inflammatory arthritis. In patients with RA, there were no significant differences in the metabolome between patients with very early or longer standing disease or between patients with RA related autoantibodies compared with patients who were seronegative. These important RA related disease features thus do not appear to influence the metabolome. Finally, some metabolites identified as biomarkers in multivariate models of RA patients’ metabolic data and CRP did not show a statistically significant univariate correlation between the metabolite concentration and CRP. This provides further evidence of the well-established importance of multivariate analysis in the field of metabolomics (45), as important relationships between metabolites and variables of interest could be overlooked through univariate analysis alone.

**Figure 8:**
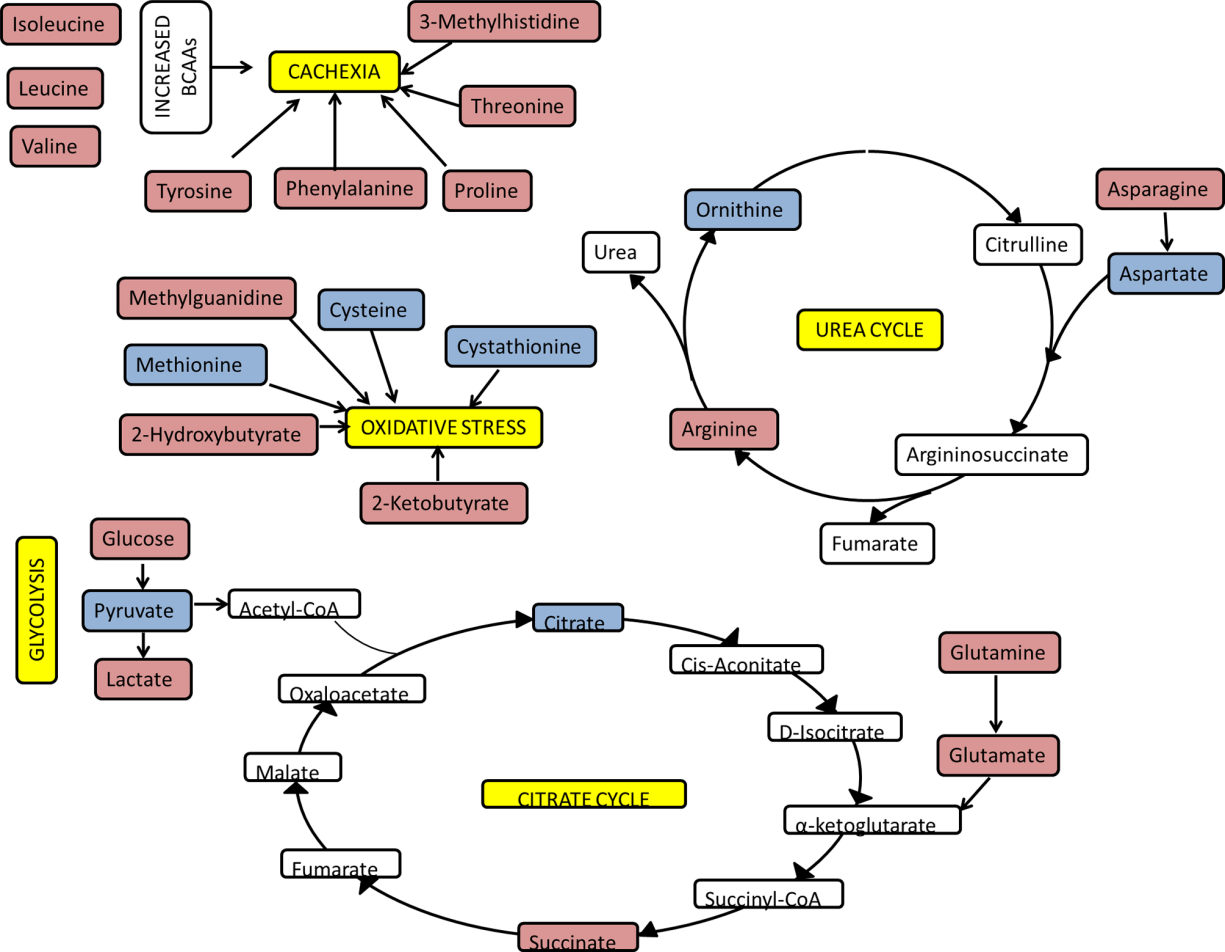
Overview of key pathways and metabolites correlating with CRP. The functional analysis of PLS-R analysis of the serum and urinary metabolome of newly presenting RA patients as assessed by ^1^H NMR spectroscopy. Red metabolites had a positive correlation with CRP and blue metabolites had a negative correlation with CRP.

**Figure 8** summarises how the metabolic changes we observed to be correlated with CRP at clinical presentation relate to increased urea cycle activity, oxidative stress, increased glycolysis, and skeletal muscle degradation related to cachexia. A positive correlation was observed between CRP and several amino acids including glutamate and phenylalanine. This could represent amino acid mobilization from protein stores such as skeletal muscle. In support of this, a positive correlation exists between CRP and 3-methylhistidine. 3-methylhistidine, the methylated analogue of histidine, is an amino acid which is present in actin and myosin (46–48). Catabolism of this complex results in 3-methylhistidine excretion and thus urinary and plasma 3-methylhistidine has been suggested as marker of skeletal muscle turnover (49–53). Leucine and valine, which are amongst the most abundant amino acids in skeletal muscle (54), also showed a positive correlation with CRP. Unlike other amino acids, a negative correlation is seen between CRP and cysteine, cystathionine, and methionine. It is possible that these amino acids are being utilised to produce glutathione. This suggests the presence of oxidative stress, as glutathione is used to reduce reactive oxygen species (55, 56).

As a result of protein catabolism there will be an increase in tissue nitrogen load. Despite the requirement for nitrogen for acute phase proteins, there appears to be a tendency to increase nitrogen excretion through upregulation of the urea cycle in proinflammatory states (57–59). Our findings suggest urea increases and aspartate decreases as CRP increases, which supports the finding of increased urea cycle activity during systemic inflammation.

A negative correlation was seen between CRP and citrate. Immune metabolic reprogramming could be responsible for this. Activation of innate immune cells, such as M1 macrophages and dendritic cells, leads to an upregulation of glycolysis and the pentose phosphate pathway, in addition to downregulation of the citrate cycle, oxidative phosphorylation and fatty acid oxidation (60). This increased glycolytic flux may represent a need to generate more ATP and other intermediates from the citrate cycle. As the citrate cycle switches from a primarily catabolic to an anabolic pathway, one consequence is the accumulation of both citrate and succinate in mitochondria (61). Citrate is transported to the cytosol and broken down to acetyl-CoA for both fatty-acid synthesis and protein acetylation, both of which have been linked to macrophage and DC activation (61). A positive correlation was seen between CRP and succinate; this has been reported previously (62). The increase in succinate, by glutamine-dependent anaplerosis, leads to HIF 1α activation and ultimately enhanced IL-1β production during inflammation (63). In addition, succinate mediated post-translational protein modification (succinylation), also perpetuates the inflammatory response.

A positive correlation was seen between CRP and glucose and lactate. Furthermore, a negative correlation was seen between CRP and pyruvate. Multiple mechanisms are likely to be responsible for the elevated glucose in proinflammatory states (26, 27, 64–69), which may serve to meet the demand for highly active immune cells. Highly active immune cells have high rates of glucose uptake and rely on glycolysis as their main form of energy production. Pyruvate is reduced to lactate even in an aerobic environment, *via* aerobic glycolysis (also known as the Warburg effect), to produce ATP rapidly (70). Lactate has significant downstream effects which propagate the inflammatory response (71–73).

Our analysis has focused on the independent analysis of the patient urine and serum metabolites and their correlation with the inflammatory process. Analysis of any relationship between the serum and urine metabolites was not possible since in this retrospective cohort we had an insufficient number of paired samples to allow a valid assessment. Nevertheless, this comparison might be useful in future studies since a correlation of a small number of metabolites has been seen in a large biobank study of urine and serum from children (74). However, in that study serum was seen to provide stronger correlates with important biological features such as age, sex, BMI and ethnicity while urine metabolites were more strongly influenced by diet. In adults, serum metabolites have been shown to be less variable than urine metabolites and so they may provide a more reliable comparator in disease states (75). However, our previous work (21), has shown that urine metabolites were able to predict responses to anti-TNF therapy in a small cohort of RA patients. Since TNF is a major driver of the inflammatory process in RA this would support our observation in the current paper that urinary metabolites do indeed reflect the inflammatory state in RA patients and as such may provide easily accessible source of useful biomarkers in RA and other inflammatory states.

## Limitations

Despite the advantages of using NMR spectroscopy to assess the metabolome, including minimal, non-destructive sample preparation, relatively low cost, good direct quantitation and high reproducibility (76), there are several important limitations. Firstly, there is low sensitivity for identifying metabolites, leading to failure to identify metabolites present at a lower concentration. Secondly, overlapping ^1^H signals can make metabolite identification difficult. For example, individual spectral peaks may be a result of a combination of metabolites rather than a single metabolite. Thus, it can be difficult to determine which of a range of possible metabolites represented within that peak is driving the association between the magnitude of that peak and the clinical variable under consideration (in this case CRP). Our use of filtered serum limited the assessment of lipid metabolism, as large proteins and lipoproteins are removed by filtration. Other confounders which are known to influence metabolism were not controlled for including comorbidities, medications, diet and time of sample collection.

## Conclusion

The PLS-R models assessing relationships between metabolite profiles and CRP have provided insight into metabolic derangements during inflammatory states such as oxidative stress, cachexia and impaired glycolytic metabolism. These findings validate a known relationship between serum metabolite profile and inflammation as measured by CRP and shows there is relationship between the urinary metabolite profile and inflammation. Serum and urine are easily accessible and easily studied by NMR spectroscopy. The focus of this paper was to examine the relationship between CRP and the serum and urinary metabolome of RA patients. Future work should examine the relationships between the metabolome and other important clinical variables including levels of pain and fatigue. Furthermore, future work should assess the impact of anti-inflammatory therapies assessing whether therapeutic response is associated with alterations in specific pathways.

## Data Availability Statement

The raw data supporting the conclusions of this article will be made available by the authors, without undue reservation.

## Ethics Statement

The studies involving human participants were reviewed and approved by Black Country Research Ethics Committee. The patients/participants provided their written informed consent to participate in this study.

## Author Contributions

GJ: data collection and manuscript composer. KS: quantified metabolites and edited manuscript. IS: data collection. AF: composed and edited manuscript. SY: composed and edited manuscript. KR: composed and edited manuscript. TA has been involved in the interpretation of the data and has reviewed, edited and approved the manuscript. All authors contributed to the article and approved the submitted version.

## Funding

This report is independent research supported by the National Institute for Health Research/Welcome Trust Clinical Research Facility at University Hospitals Birmingham NHS Foundation Trust. GJ was funded through a fellowship from the MRC/ARUK Centre for Musculoskeletal Ageing Grant MR/K00414X/1). This work and KS PhD studentship was supported by the Arthritis Research UK Rheumatoid Arthritis Pathogenesis Centre of Excellence (20298) and the European Community’s Collaborative project FP7-HEALTH-F2-2012-305549 ‘Euro-TEAM’. AF was supported by an Arthritis Research UK Clinician Scientist Award (18547). AF and KR are supported by the NIHR Birmingham Biomedical Research Center (BRC-1215-20009). This work was supported in part by NPRP grant # [NPRP 7 - 959 - 3 - 246] from the Qatar National Research Fund (a member of Qatar Foundation).

## Conflict of Interest

The authors declare that the research was conducted in the absence of any commercial or financial relationships that could be construed as a potential conflict of interest.

## Publisher’s Note

All claims expressed in this article are solely those of the authors and do not necessarily represent those of their affiliated organizations, or those of the publisher, the editors and the reviewers. Any product that may be evaluated in this article, or claim that may be made by its manufacturer, is not guaranteed or endorsed by the publisher.

## Supplementary Material

Supplementary Figure 1 PLS-R analysis showing the relationship between metabolic data derived from RA patients and ESR. **(A)** Using the full 590 serum metabolite binned data (n = 120) there was a correlation between metabolite data and ESR on PLS-R analysis (r^2^ = 0.15, 5 LV, p = 0.013). **(B)** Using the full 900 NMR urinary metabolite bins for RA patients (n = 79) there was a correlation between metabolite profile and ESR (r^2^ = 0.19, 5 LV, p = 0.014).

Supplementary Figure 2 OPLS-DA of RA patients’ metabolome by autoantibody status and symptom duration. **(A)** OPLS-DA plot of serum metabolic data (n = 126, blue is seronegative*, green is seropositive*; 1 + 0+0 LV, p = 1) showing no separation between the two groups. **(B)** OPLS-DA plot of urinary metabolic data (n = 83, blue is seronegative*, green is seropositive*; 1 + 0+0 LV, p = 1) showing no separation between the two groups. **(C)** OPLS-DA plot of serum metabolic data (n = 126, blue is symptom duration of ≤12 weeks, green is symptom duration of >12weeks; 1 + 0+0 LV, p = 0.556) showing no separation between the two groups. **(D)** OPLS-DA plot of urinary metabolic data (n = 83, blue is symptom duration of ≤12 weeks, green is symptom duration of >12weeks; 1 + 1+0 LV, p = 1) showing no separation between the two groups. *seropositive for either ACPA or RF or both.

Supplementary Figure 3 PLS-R analysis showing the relationship between metabolic data derived from UA patients and CRP. **(A)** UA patients have a statistically significant relationship between serum metabolite profile and CRP (n = 41, 98 NMR bins post forward selection, r^2^ = 0.7209, 9 LV, p < 0.001). **(B)** UA patients have a statistically significant relationship between urinary metabolite profile and CRP (n = 25, 90 NMR bins post forward selection, r^2^ = 0.6117, 8 LV, p = 0.025).
