# Supplementary figures and images for: Relationship Between Inflammation and Metabolism in Patients With Newly Presenting Rheumatoid Arthritis
Source: Front Immunol. 2021 Sep 28;12:676105. doi: 10.3389/fimmu.2021.676105 (PMC8507469; doi:10.3389/fimmu.2021.676105)

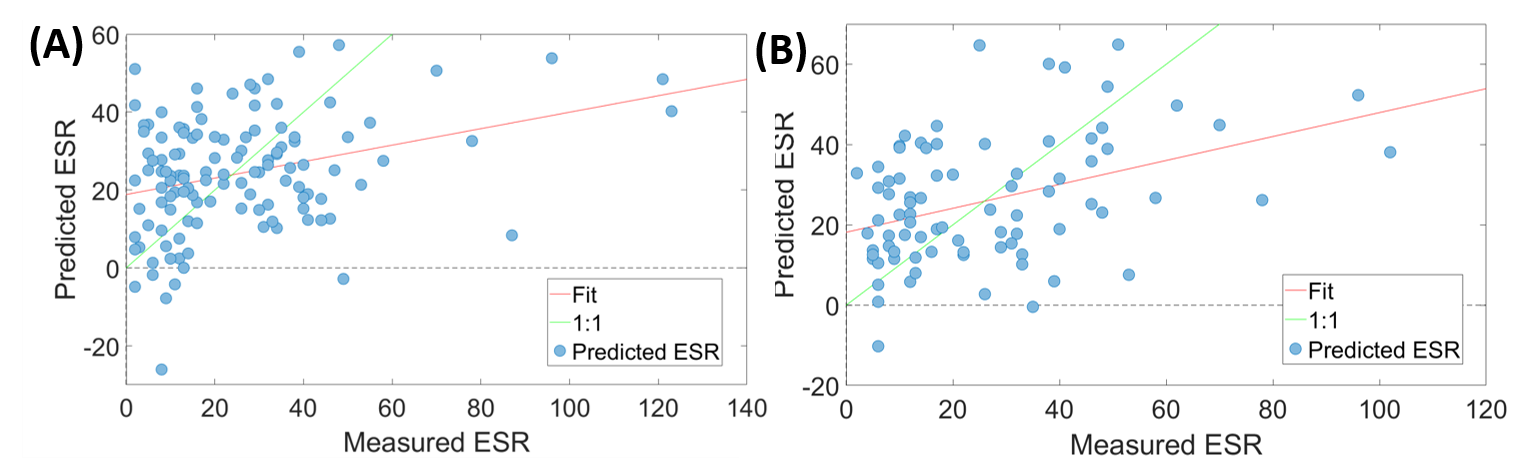

Supplement: Supplementary Figure 1 — PLS-R analysis showing the relationship between metabolic data derived from RA patients and ESR. (A) Using the full 590 serum metabolite binned data (n = 120) there was a correlation between metabolite data and ESR on PLS-R analysis (r2 = 0.15, 5 LV, p = 0.013). (B) Using the full 900 NMR urinary metabolite bins for RA patients (n = 79) there was a correlation between metabolite profile and ESR (r2 = 0.19, 5 LV, p = 0.014). [file Image_1.tif]

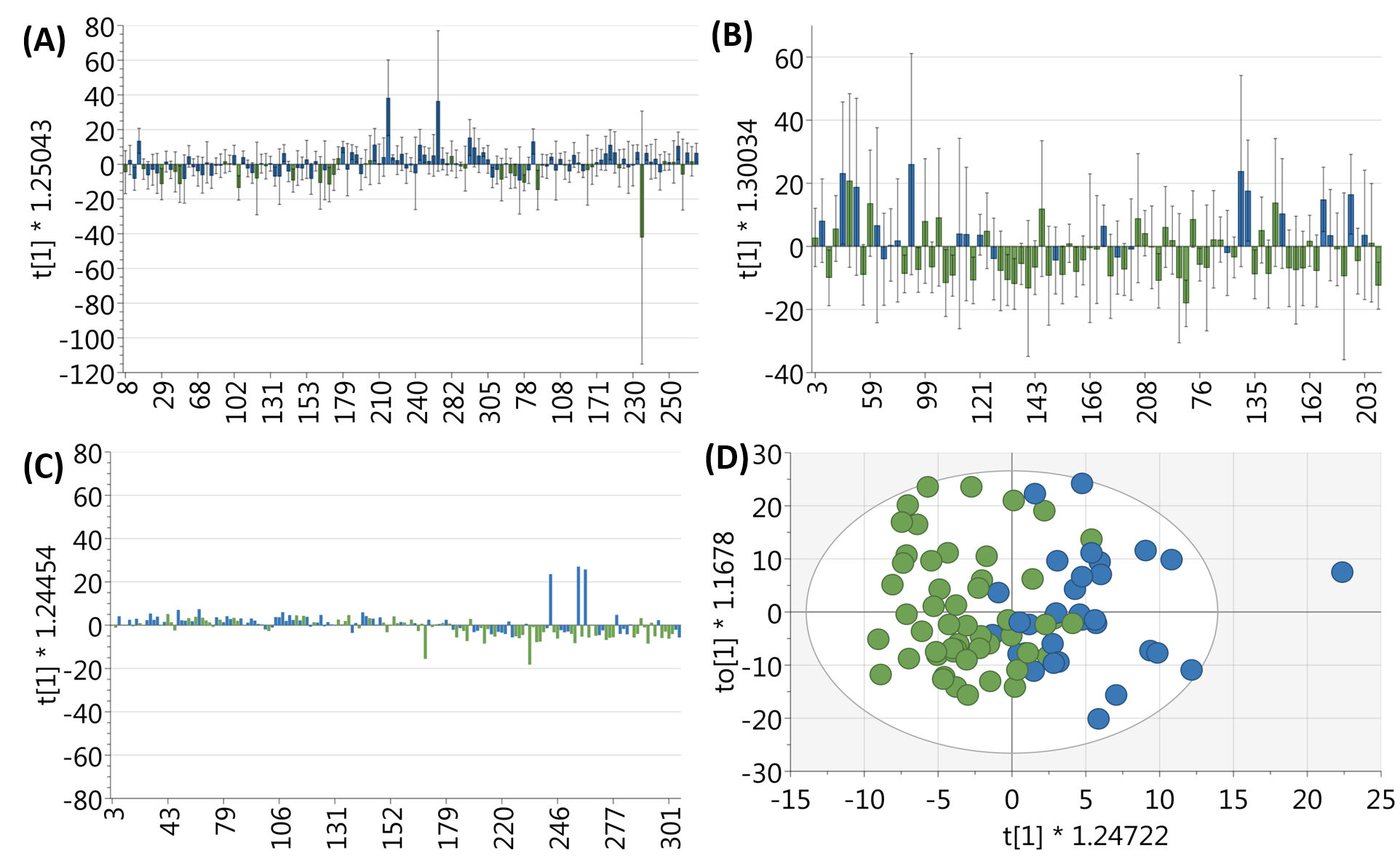

Supplement: Supplementary Figure 2 — OPLS-DA of RA patients’ metabolome by autoantibody status and symptom duration. (A) OPLS-DA plot of serum metabolic data (n = 126, blue is seronegative*, green is seropositive*; 1 + 0+0 LV, p = 1) showing no separation between the two groups. (B) OPLS-DA plot of urinary metabolic data (n = 83, blue is seronegative*, green is seropositive*; 1 + 0+0 LV, p = 1) showing no separation between the two groups. (C) OPLS-DA plot of serum metabolic data (n = 126, blue is symptom duration of ≤12 weeks, green is symptom duration of >12weeks; 1 + 0+0 LV, p = 0.556) showing no separation between the two groups. (D) OPLS-DA plot of urinary metabolic data (n = 83, blue is symptom duration of ≤12 weeks, green is symptom duration of >12weeks; 1 + 1+0 LV, p = 1) showing no separation between the two groups. *seropositive for either ACPA or RF or both. [file Image_2.tif]

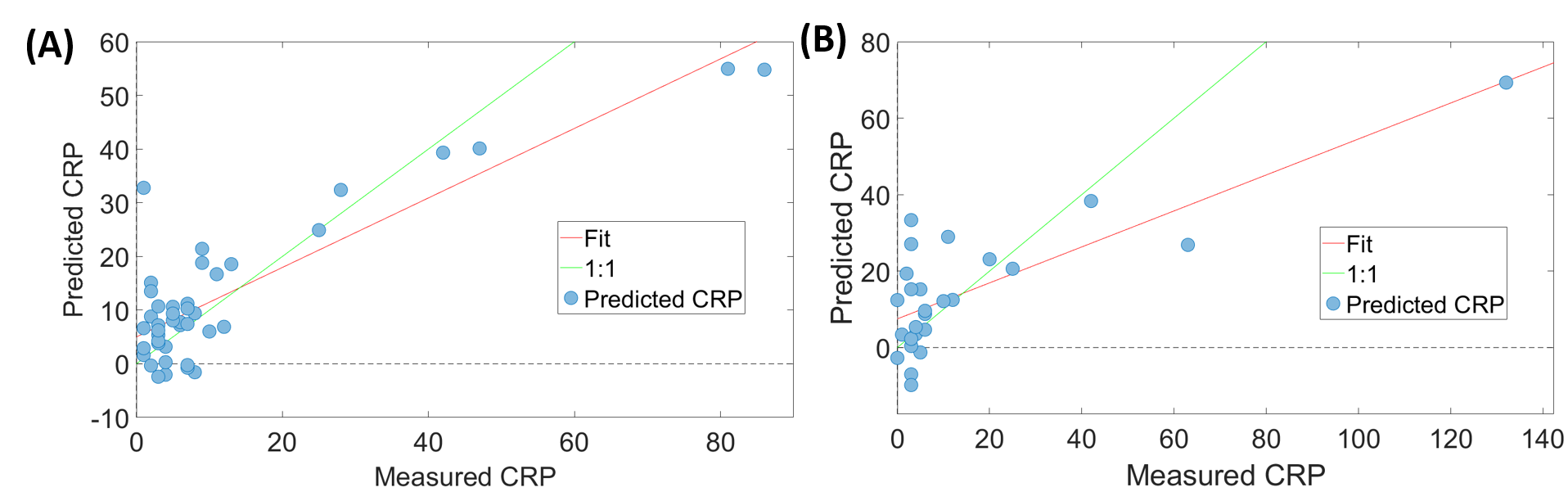

Supplement: Supplementary Figure 3 — PLS-R analysis showing the relationship between metabolic data derived from UA patients and CRP. (A) UA patients have a statistically significant relationship between serum metabolite profile and CRP (n = 41, 98 NMR bins post forward selection, r2 = 0.7209, 9 LV, p < 0.001). (B) UA patients have a statistically significant relationship between urinary metabolite profile and CRP (n = 25, 90 NMR bins post forward selection, r2 = 0.6117, 8 LV, p = 0.025). [file Image_3.tif]
